# Supplementary material for: Predicting visual function by interpreting a neuronal wiring diagram
Source: Nature. 2024 Oct 2;634(8032):113–23. doi: 10.1038/s41586-024-07953-5 (PMC11446822; doi:10.1038/s41586-024-07953-5)
Supplement: Supplementary file 6 — CRF and ERF predictions for individual TmY4 and TmY9 cells. Analogous to Supplementary Data 3, but for TmY target types. Shown are the top four monosynaptic pathways, the strongest pathway passing through each of the top ten intermediary types (ranking from Extended Data Fig. 7), and the trisynaptic pathway Tm1–TmY–Dm3–TmY (see the section entitled Prediction of spatial normalization). [file 41586_2024_7953_MOESM6_ESM.zip › DataS4/TmY9q/720575940628923330.pdf]

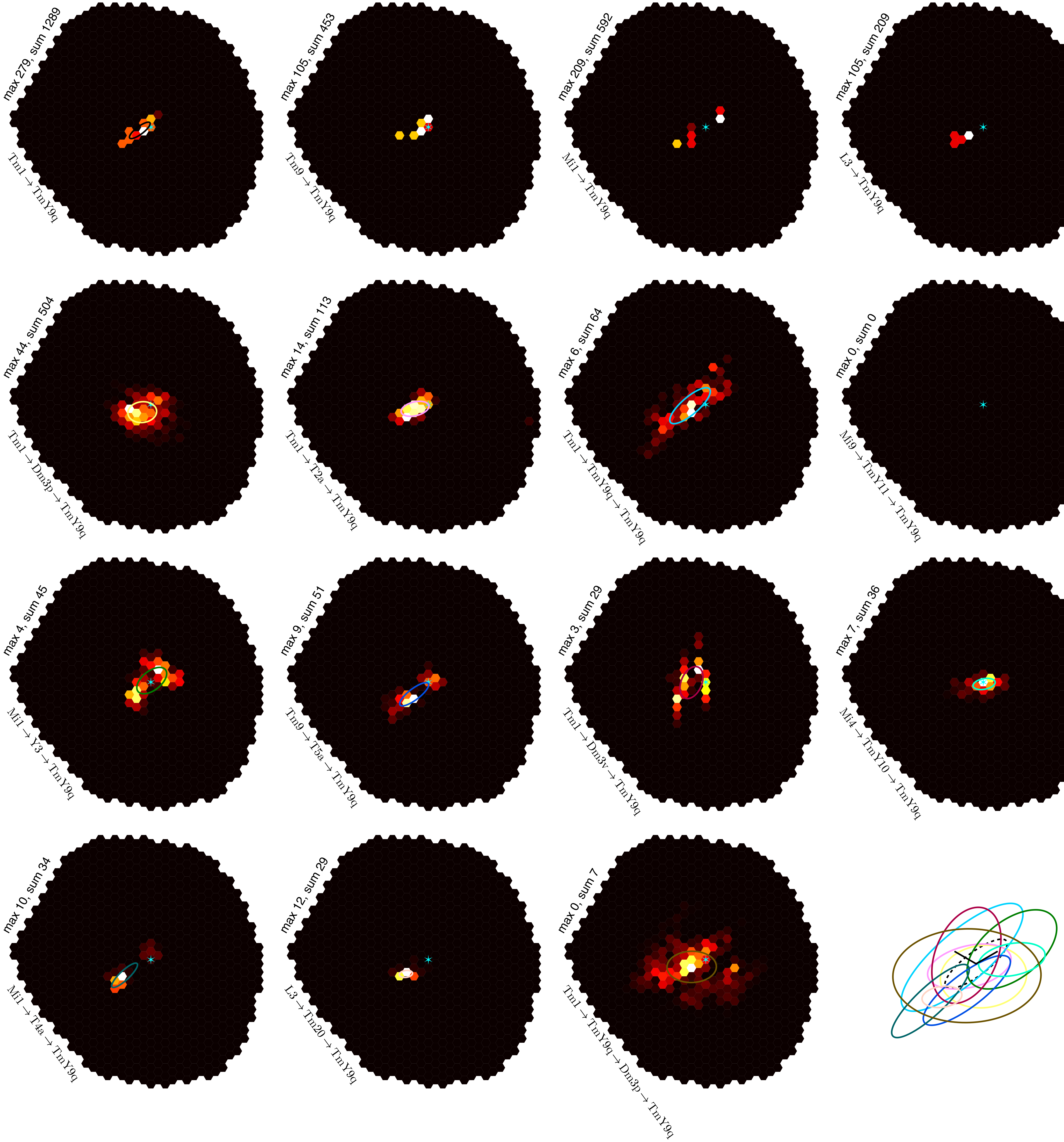
max 279, sum 1289
Tm1 → TmY9q
max 105, sum 453
Tm9 → TmY9q
max 209, sum 592
Mi1 → TmY9q
max 105, sum 209
L3 → TmY9q
max 44, sum 504
Tm1 → Dm3p → TmY9q
max 14, sum 113
Tm1 → T2a → TmY9q
max 6, sum 64
Tm1 → TmY9q → TmY9q
max 0, sum 0
Mi9 → TmY11 → TmY9q
max 4, sum 45
Mi1 → Y3 → TmY9q
max 9, sum 51
Tm9 → T5a → TmY9q
max 3, sum 29
Tm1 → Dm3v → TmY9q
max 7, sum 36
Mi4 → TmY10 → TmY9q
max 10, sum 34
Mi1 → T4a → TmY9q
max 12, sum 29
L3 → Tm20 → TmY9q
max 0, sum 7
Tm1 → TmY9q → Dm3p → TmY9q
